# Draft Genome Sequences of the Nitrate-Dependent Iron-Oxidizing Proteobacteria *Acidovorax* sp. Strain BoFeN1 and Paracoccus pantotrophus Strain KS1

**DOI:** 10.1128/MRA.01050-18

**Published:** 2018-09-13

**Authors:** A. Price, M. C. Macey, J. Miot, K. Olsson-Francis

**Affiliations:** a Faculty of Science, Technology, Engineering and Mathematics, The Open University, Milton Keynes, United Kingdom; b Institut de Minéralogie, de Physique des Matériaux et de Cosmochimie, Muséum National d’Histoire Naturelle, Université Pierre et Marie Curie–Sorbonne Universités, CNRS UMR 7590, IRD 206, Paris, France; Louisiana State University

## Abstract

The draft genomes of the nitrate-dependent iron-oxidizing bacteria *Acidovorax* sp. strain BoFeN1 and Paracoccus pantotrophus strain KS1 are presented. These genomes supply supporting data to investigations of the mechanisms underlying this anaerobic form of microbial biogeochemical iron cycling.

## Abstract (precis)

The draft genomes of the nitrate-dependent iron-oxidizing bacteria *Acidovorax* sp. strain BoFeN1 and Paracoccus pantotrophus strain KS1 are presented.

## ANNOUNCEMENT

*Acidovorax* sp. strain BoFeN1 and Paracoccus pantotrophus strain KS1 have both demonstrated the capability to perform nitrate-dependent Fe^2+^ oxidation (NDFO), whereby Fe^3+^ precipitates are formed during microbial nitrate reduction (1, 2). NDFO is a geomicrobiological process which has been recognized for the past 2 decades (3) and is responsible for the production of Fe^3+^-bearing minerals in anaerobic, circumneutral, reducing environments under conditions similar to those that were prevalent on the early Earth and ancient Mars (1, 4–6). Despite this potential importance in the early history of life, the putative underlying mechanisms are largely unconfirmed experimentally (7), despite some advances (8, 9).

*Acidovorax* sp. strain BoFeN1 is a Gram-negative, rod-shaped bacterium belonging to the family *Comamonadaceae* in the order *Burkholderiales* of the class *Betaproteobacteria*. *Acidovorax* sp. strain BoFeN1 was isolated from littoral sediments of Lake Constance on the Germany-Switzerland border and assigned to the genus *Acidovorax* based on 16S rRNA gene sequence analysis (1). This isolate was donated by Jennyfer Miot of the Mineralogy, Material Physics and Cosmochemistry Institute (IMPMC) in Paris, France.

Paracoccus pantotrophus strain KS1 is a Gram-negative, motile coccoid bacterium belonging to the family *Rhodobacteraceae* in the order *Rhodobacterales*, in the class *Alphaproteobacteria*. Paracoccus pantotrophus strain KS1 was isolated from soil at Kew Gardens in the United Kingdom and was originally reported as a member of the genus *Thiobacillus* but was later reclassified to the genus *Paracoccus* based on the 16S rRNA gene sequence (10, 11). This strain was purchased as a live culture (DSM 11072) from the German Collection of Microorganisms and Cell Cultures (DSMZ) in Leibniz, Germany.

The two strains were maintained in pure culture in aerobic nutrient medium (5.0 g liter^−1^ peptone and 3.0 g liter^−1^ meat extract) at 30°C prior to DNA extraction and genome sequencing, which was performed by MicrobesNG (https://microbesng.uk/) using the Illumina MiSeq platform with 2 × 250-bp paired-end reads. Trimmed reads were produced using Trimmomatic version 0.30 (http://www.usadellab.org/cms/?page=trimmomatic) with a sliding window quality cutoff of Q15, and *de novo* assembly was performed using SPAdes version 3.7 (http://cab.spbu.ru/software/spades/) (12, 13). Coverage of 30× was achieved during sequencing, and genome annotation was performed using the RAST annotation server version 2.0 (http://rast.nmpdr.org/rast.cgi) via the classic RAST pipeline (14). The draft genome of strain *Acidovorax* sp. strain BoFeN1 consisted of 4.06 Mb in 184 contigs (*N*_50_ = 37,384), with a GC content of 63.77%. A total of 3,794 protein-coding sequences were predicted from the annotated genome, with 50 tRNAs identified using the ARAGORN version 1.1 tRNA detection program (http://mbio-serv2.mbioekol.lu.se/ARAGORN/) (15). The closest match for the 16S rRNA gene was identified in the GenBank database, by using the BLASTN program (https://blast.ncbi.nlm.nih.gov/Blast.cgi), as Acidovorax defluvii strain BSB411 (99% similarity; GenBank accession number NR_026506). The draft genome of Paracoccus pantotrophus strain KS1 consisted of 4.16 Mb in 227 contigs (*N*_50_ = 36,889), with a GC content of 67.69%. A total of 3,941 protein-coding sequences and 54 tRNAs were predicted from the annotated genome. The nearest 16S rRNA gene sequence was identified using BLASTN, with a best match as Paracoccus pantotrophus (100%; accession number AB098590).

Neither genome contained a dedicated ferroxidase, suggesting that NDFO in these strains is not a direct enzymatic process to acquire electrons. Full denitrification pathways were confirmed in both strains by the presence of nitrogen species reductase genes (*nar*, *nir*, *nor*, and *nos*), with additional genes for a periplasmic nitrate reductase (Nap) in the Paracoccus pantotrophus strain KS1 genome.

### Data availability.

DDBJ/ENA/GenBank accession numbers have been assigned for *Acidovorax* sp. strain BoFeN1 (QOZT00000000) and *Paracoccus pantotrophus* strain KS1 (QOZU00000000). Raw sequencing reads for *Acidovorax* sp. strain BoFeN1 (SRP157586) and *Paracoccus pantotrophus* strain KS1 (SRP157588) are available in the NCBI Sequence Read Archive.

## References

[1] Kappler A, Schink B, Newman DK 2005 Fe(III) mineral formation and cell encrustation by the nitrate-dependent Fe(II)-oxidizer strain BoFeN1. Geobiology 3:235–245. doi:10.1111/j.1472-4669.2006.00056.x.

[2] Kumaraswamy R, Sjollema K, Kuenen G, Van Loosdrecht M, Muyzer G 2006 Nitrate-dependent [Fe(II)EDTA]^2−^ oxidation by *Paracoccus ferrooxidans* sp. nov., isolated from a denitrifying bioreactor. Syst Appl Microbiol 29:276–286. doi:10.1016/j.syapm.2005.08.001. 16682296

[3] Straub KL, Benz M, Schink B, Widdel F 1996 Anaerobic, nitrate-dependent microbial oxidation of ferrous iron. Appl Environ Microbiol 62:1458–1460. 16535298 10.1128/aem.62.4.1458-1460.1996 PMC1388836

[4] Miot J, Etique M 2016 Formation and transformation of iron-bearing minerals by iron (II)-oxidizing and iron (III)-reducing bacteria, p. 53–98. *In* Faivre D (ed), Iron oxides: from nature to applications. Wiley-VCH, Weinheim, Germany.

[5] Ilbert M, Bonnefoy V 2013 Insight into the evolution of the iron oxidation pathways. Biochim Biophys Acta 1827:161–175. doi:10.1016/j.bbabio.2012.10.001. 23044392

[6] Price A, Pearson VK, Schwenzer SP, Miot J, Olsson-Francis K 2018 Nitrate-dependent iron oxidation: a potential mars metabolism. Front Microbiol 9:513. doi:10.3389/fmicb.2018.00513. 29616015 PMC5869265

[7] Carlson H, Clark I, Melnyk R, Coates J 2012 Toward a mechanistic understanding of anaerobic nitrate-dependent iron oxidation: balancing electron uptake and detoxification. Front Microbiol 3:57. doi:10.3389/fmicb.2012.00057. 22363331 PMC3282478

[8] Carlson HK, Clark IC, Blazewicz SJ, Iavarone AT, Coates JD 2013 Fe(II) oxidation is an innate capability of nitrate-reducing bacteria that involves abiotic and biotic reactions. J Bacteriol 195:3260–3268. doi:10.1128/JB.00058-13. 23687275 PMC3697641

[9] Miot J, Remusat L, Duprat E, Gonzalez A, Pont S, Poinsot M 2015 Fe biomineralization mirrors individual metabolic activity in a nitrate-dependent Fe(II)-oxidizer. Front Microbiol 6:879. doi:10.3389/fmicb.2015.00879. 26441847 PMC4562303

[10] Jordan SL, Kraczkiewicz-Dowjat AJ, Kelly DP, Wood AP 1995 Novel eubacteria able to grow on carbon disulfide. Arch Microbiol 163:131–137. 10.1007/s002030050492 9382702

[11] Jordan SL, McDonald IR, Kraczkiewicz-Dowjat AJ, Kelly DP, Rainey FA, Murrell JC, Wood AP 1997 Autotrophic growth on carbon disulfide is a property of novel strains of *Paracoccus denitrificans*. Arch Microbiol 168:225–236. doi:10.1007/s002030050492. 9382702

[12] Bolger AM, Lohse M, Usadel B 2014 Trimmomatic: a flexible trimmer for Illumina sequence data. Bioinformatics 30:2114–2120. doi:10.1093/bioinformatics/btu170. 24695404 PMC4103590

[13] Bankevich A, Nurk S, Antipov D, Gurevich AA, Dvorkin M, Kulikov AS, Lesin VM, Nikolenko SI, Pham S, Prjibelski AD, Pyshkin AV, Sirotkin AV, Vyahhi N, Tesler G, Alekseyev MA, Pevzner PA 2012 SPAdes: a new genome assembly algorithm and its applications to single-cell sequencing. J Comput Biol 19:455–477. doi:10.1089/cmb.2012.0021. 22506599 PMC3342519

[14] Aziz RK, Bartels D, Best AA, DeJongh M, Disz T, Edwards RA, Formsma K, Gerdes S, Glass EM, Kubal M, Meyer F, Olsen GJ, Olson R, Osterman AL, Overbeek RA, McNeil LK, Paarmann D, Paczian T, Parrello B, Pusch GD, Reich C, Stevens R, Vassieva O, Vonstein V, Wilke A, Zagnitko O 2008 The RAST server: Rapid Annotations using Subsystems Technology. BMC Genomics 9:75. doi:10.1186/1471-2164-9-75. 18261238 PMC2265698

[15] Laslett D, Canback B 2004 ARAGORN, a program to detect tRNA genes and tmRNA genes in nucleotide sequences. Nucleic Acids Res 32:11–16. doi:10.1093/nar/gkh152. 14704338 PMC373265

